# Annurca Apple Polyphenols Protect Murine Hair Follicles from Taxane Induced Dystrophy and Hijacks Polyunsaturated Fatty Acid Metabolism toward β-Oxidation

**DOI:** 10.3390/nu10111808

**Published:** 2018-11-20

**Authors:** Gennaro Riccio, Eduardo Sommella, Nadia Badolati, Emanuela Salviati, Sara Bottone, Pietro Campiglia, Monica Dentice, Gian Carlo Tenore, Mariano Stornaiuolo, Ettore Novellino

**Affiliations:** 1 Department of Pharmacy, University of Naples Federico II. Via Montesano 49, 80149 Naples, Italy; genriccio@gmail.com (G.R.); badolatin@gmail.com (N.B.); sara.bottone@unina.it (S.B.); giancarlo.tenore@unina.it (G.C.T.); 2 Department of Pharmacy, School of Pharmacy, University of Salerno, Via Giovanni Paolo II 132, I-84084 Fisciano, Italy; esommella@unisa.it (E.S.); esalviati@unisa.it (E.S.); pcampiglia@unisa.it (P.C.); 3 PhD Program in Drug Discovery and Development, University of Salerno, Via Giovanni Paolo II 132, I-84084 Fisciano, Italy; 4 Department of Clinical Medicine and Surgery, University of Naples Federico II, Via Pansini 5, 80149 Naples, Italy; monica.dentice@unina.it

**Keywords:** nutraceuticals, apple polyphenols, PUFA, Prostaglandins F2, taxanes, chemotherapy induced alopecia

## Abstract

Chemotherapy-induced alopecia (CIA) is a common side effect of conventional chemotherapy and represents a major problem in clinical oncology. Even months after the end of chemotherapy, many cancer patients complain of hair loss, a condition that is psychologically difficult to manage. CIA disturbs social and sexual interactions and causes anxiety and depression. Synthetic drugs protecting from CIA and endowed with hair growth stimulatory properties are prescribed with caution by oncologists. Hormones, growth factors, morphogens could unwontedly protect tumour cells or induce cancer cell proliferation and are thus considered incompatible with many chemotherapy regimens. Nutraceuticals, on the contrary, have been shown to be safe and effective treatment options for hair loss. We here show that polyphenols from *Malus Pumila Miller* cv Annurca are endowed with hair growth promoting activity and can be considered a safe alternative to avoid CIA. In vitro, Annurca Apple Polyphenolic Extract (AAE) protects murine Hair Follicles (HF) from taxanes induced dystrophy. Moreover, in virtue of its mechanism of action, AAE is herein proven to be compatible with chemotherapy regimens. AAE forces HFs to produce ATP using mitochondrial β-oxidation, reducing Pentose Phosphate Pathway (PPP) rate and nucleotides production. As consequence, DNA replication and mitosis are not stimulated, while a pool of free amino acids usually involved in catabolic reactions are spared for keratin production. Moreover, measuring the effect exerted on Poly Unsaturated Fatty Acid (PUFA) metabolism, we prove that AAE promotes hair-growth by increasing the intracellular levels of Prostaglandins F2α (PGF2α) and by hijacking PUFA catabolites toward β-oxidation.

## 1. Introduction

The process of extension of hair shaft in humans occurs in a specific phase of the hair follicle (HF) cycle, the anagen. Each anagen phase can last years and is interspersed with short lasting phases of degeneration (catagen) and resting, (telogen) [1]. Hair shaft extension is sustained by highly proliferating cells of the hair matrix (HMs). These differentiate into several cell lineages, (among these cells of the outer (ORS) and inner (IRS) root sheaths) all differently specialized in the production and modification of hair keratins [1,2,3,4]. 

In virtue of their high rate of proliferation, healthy HFs cells are easy victims of chemotherapy agents [5,6,7,8,9]. Anticancer drugs impair mitosis and induce premature apoptosis, HF miniaturization and hair bulb dystrophy [10,11]. The resulting chemotherapy-induced alopecia (CIA) is one of the most common side events caused by conventional cytotoxic chemotherapy and represents a major problem in clinical oncology [12]. Chemotherapy regimens based on doxorubicin, anthracycline, cyclophosphamide and taxanes lead to massive apoptosis in HM keratinocytes followed by a transitory or even irreversible hair loss. In particular, patients in taxane regimen undergo irreversible alopecia more than those treated with other classes of chemotherapy agents [13]. 

CIA is not life-threatening, however it represents a psychologically difficult event to manage, especially for women. The stigma of CIA are distressing, disturb social interactions, cause loss in self-confidence and self-esteem, alteration in sexuality and induce anxiety and depression [14].

Up to today, no treatment is currently available to prevent or retard CIA. This gap is also due to an understandable reluctance of oncologists. Priority for the physicians is and must remain the eradication of cancer cells and the avoidance of tumour relapses. The usage of agents protecting HFs from CIA could unwontedly stimulate cancer cell proliferation, an event definitely more dangerous than hair loss. In virtue of their ability to induce proliferation in virtually any healthy and cancer cell, hormones endowed with hair stimulating activity, hair growth factors, HF morphogens (like the Wnt/β-catenin pathway agonists Valproic acid, Lithium Chloride and Wnt7a) [15,16,17] are all considered incompatible with chemotherapy regimens. 

Recently, nutritional and antioxidant therapies have been shown to be effective options for hair loss and, in virtue of their safety, are becoming popular over-the-counter products. We have shown that nutraceuticals obtained from *Malus Pumila Miller* cv Annurca, an apple native to Southern Italy and highly enriched in Procyanidin B2, are able to promote hair growth and keratin expression in humans [18,19]. In a clinical trial describing the effect of Annurca Apple polyphenolic extract (AAE) on hair growth in healthy subjects, the consumption of AAE exerted significant results in terms of hair number, hair weight and keratin content [20]. Recently, we have started elucidating the molecular details behind the hair growth promoting effect of AAE and have shown that this does not result from stimulation of HF cell proliferation [21]. Topical treatment with a cosmetic foam containing AAE strongly affects, in vivo, the overall red-ox environment of healthy HF cells forcing them through a drastic metabolic switch. While HFs normally produce ATP via aerobic glycolysis, glutaminolysis and Pentose Phosphate Pathway (PPP) [22], AAE forces HFs to produce ATP using mitochondrial β-oxidation. In the presence of active mitochondria, HFs thus spare amino acids (mainly Glutamine, Glycine, Arginine and Cysteine) from oxidation and use them for keratin production. Moreover, as consequence of the metabolic switch, the syntheses of nitrogen containing bases and of deoxy-nucleotides in HFs are inhibited by AAE. The absence of metabolites necessary for DNA replication, RNA production and mitosis confirms that AAE hair growth stimulation does not rely on induction of cell proliferation.

Herein, we analyse new aspects of the molecular mechanism behind AAE activity by analysing how the apple extract affects the metabolism of PUFA, a class of lipids and signalling molecules involved in HFs homeostasis [23,24,25,26]. PUFA signalling influences as well response to chemotherapy and is extremely important in terms of immunosuppression, tumour growth and tumour relapse [27,28]. Arachidonic Acid (ARA), a ω-6 PUFA, significantly promotes hair shaft elongation and proliferation of HM keratinocytes in ex-vivo HF cultures [29,30]. Nowadays, clinical trials are running to test the hair growth promotion induced by latanoprost [31] and isopropyl unoprostone [32], both analogues of Prostaglandin F2α, one of the major metabolite of ARA. 

We treated C57BL/6 mice with a cosmetic foam containing AAE (topically and for 4 weeks) to then extract their HFs and analyse their metabolome by Direct Infusion Fourier Transform-ion cyclotron resonance mass spectrometry (FT-ICR-MS), a technique endowed with ultra-high mass accuracy and resolution [33]. We prove that AAE increases the intracellular levels of the growth promoting prostanoid Prostaglandins F2α (PGF2α). On the contrary, AAE reduces the intracellular levels of PUFA epoxides stimulating their conversion into the corresponding inactive diols and promoting their usage as β-oxidation substrates. 

We finally use an in vitro assay and prove for the first time that AAE can inhibit taxane induced dystrophy in ex-vivo murine HFs. The increase in keratin production, the lack of stimulation of cell proliferation and mitosis, the modulation of lipid molecules all suggest that AAE could represent a safe nutraceutical for hair growth and an interesting alternative to synthetic drugs for treating CIA. In taxane-containing chemotherapy regimen, AAE could be even more indicated, considering the selectivity of this chemotherapy agent for mitotic cells. 

## 2. Materials and Methods 

### 2.1. Reagents and Nutraceuticals 

AAE Annurca Apple Extract AnnurtriComplex (industrial procyanidinic extract of Annurca apple PGI (Protected Geographical Indication) (*Malus Pumila Miller* cv Annurca)) was produced by MB-Med (Turin, Italy) as already described [34]. For in vitro experiments it was dissolved in DMSO to achieve a stock concentration of 30 mg/mL and stored at −20 °C in the dark. Composition of AAE cosmetic foam: AnnurtriComplex 6% (*w*/*v*), water, glycerin, decylglucoside, polysorbate, maltodextrin, potassium sorbate, sodium benzoate, silica. The Placebo foam was formulated identically but did not contain AnnurtriComplex. DAPI was from Sigma Chemical Co. (St. Louis, MO, USA) was used as already described [35]. Paclitaxel and Docetaxel were kindly provided by the Hospital Pharmacy of Istituto Nazionale Tumori-IRCCS-FondazioneG. Pascale. 

### 2.2. Animals 

#### 2.2.1. Animals for Ex-Vivo Culturing of Murine HFs

Wild-type C57BL/6 mice were used for the ex vivo experiments. Only male animals were used in this study. All animal experiments were performed in compliance with ethical guidelines and approved by the University of Naples Federico II. 12 weeks old mice (postnatal day 84) were sacrificed and their dorsal skin were immediately excised and immersed in Phosphate Buffer Saline (PBS). 1 cm^2^ of skin biopsies, were rinsed in PBS and located in 6 multiwell plates. Biopsies were cultivated in 1 mL of Dulbecco Modified Eagle Medium (41965-039, GIBCO, Thermo Fisher Scientific, Waltham, MA, USA) supplemented with 10% FBS (10270, GIBCO), Glutamine (35050-061, GIBCO), Penicillin and Streptomycin (15070-063, GIBCO). Tissues were incubated for 8 days in a Cell Culture incubator at 37 °C, supplemented with 5% CO_2_. When indicated, Paclitaxel (700 nM), Docetaxel (700 nM) and/or AAE (400 mg/L) were added to the culture medium. The medium was replenished every 3 days. At the end of the incubation, tissues were rinsed three times in DMEM and fixed in 4% formaldehyde diluted in PBS (pH 7.4). Nuclei were stained with DAPI and visualized under a fluorescent microscope as already described [35]. Scanning Electron Microscopy (SEM) and Energy Dispersive X-Ray (EDX) analysis of hair shafts were performed with a bench top Phenom XL (Alfatest, Milan, Italy) following manufacturer instructions and as already described [36]. 

#### 2.2.2. Animals for In-Vivo Experiments

Wild-type C57BL/6 mice (7 weeks old, postnatal day 49) were used in all experiments to test the effect of cosmetic foam containing AAE. All animals received human care and were maintained in separate cages at 22 °C–24 °C and fed a general rodent diet. Differently from other published protocols, here animals were left unshaved and received a topical treatment with 2 cm^3^ of the indicated cosmetic foam for 4 weeks. Only male animals were used in this study. All animal experiments were performed in compliance with ethical guidelines and approved by the University of Naples Federico II.

### 2.3. Metabolite Extraction from Murine Tissues

Immediately after excision, tissues were rinsed and kept in PBS. Hair shafts were plucked out with a sterile tweezers and immediately covered with a solution of PBS at R.T. To allow detachment of HF cells, plucked HFs were incubated for 15 min in PBS supplemented with 5 mM EDTA. Hair shafts were removed with a cell strainer and HF cells were centrifuged for 5 min at 500 rpm. The cell pellets were washed twice in PBS to be then homogenized in 1 mL of pre-chilled methanol/water 1:1 solution containing 10 nmol of internal standard and finally centrifuged at 10,000× *g* for 10 min at 4 °C [37]. The resulting supernatants were collected and transferred into new Eppendorf tubes and stored at −80 °C. 

### 2.4. Mass Spectrometry-Based Metabolomic, Statistics and Analysis

Analyses were performed in direct infusion following a previous protocol [33,38] employing a Hamilton syringe (250 μL) at a flow rate of 2 μL/min. Data were acquired on a SolariX XR 7T (Bruker Daltonics, Bremen, Germany). The instrument was tuned with a standard solution of sodium trifluoroacetate. Mass Spectra were recorded in broadband mode in the range 100–1500 m/z, with an ion accumulation of 20 ms, with 32 scans using 2 million data points (2M). Nebulizing (N_2_) and drying gases (air) were set at 1 and 4 mL/min, respectively, with a drying temperature of 200 °C. Both positive and negative ESI ionization were employed. Five replicates of each injection were carried out. The instrument was controlled by Bruker FTMS Control, MS spectra were elaborated with Compass Data Analysis version 4.2 (Bruker, New York, NY, USA), identification of compounds based on accurate MS measurements was performed by Compound Crawler ver. 3.0 and Metaboscape 3.0 (Bruker). Metabolites signals were normalized using internal standards. Comparisons and differences were analysed for statistical significance by two-way Anova test and Bonferroni post-tests analysis. All graphs, bars or lines indicate mean and error bars indicate standard error of the mean (s.e.m.). Furthermore, Partial Least Square Discriminant Analysis (PLS-DA) was used as classification model. All graphs, bars or lines indicate mean and error bars indicate standard error of the mean (s.e.m.). Statistical analysis was performed using Statistica software (StatSoft, Tulsa, OK, USA) and Minitab (Minitab Inc., State College, PA, USA).

## 3. Results

### 3.1. Topical Treatment with AAE Alters PUFAs Metabolism in Murine HFs 

We started implementing our recently published metabolomic analysis [21] by investigating the effect exerted by AAE on the metabolism of PUFAs. These lipids are present in an esterified form in all mammalian cell membranes and are released for signalling purposes by the action of Phospholipase A2. Free PUFA are made available as a substrate for the intercellular biosynthesis of various metabolites through the action of cyclooxygenases (COX1, COX2), lipoxygenases (LOX5, LOX12/15) and cytochrome P-450 (CYP). An increase in PUFAs after AAE treatment could have been indeed represented a likely explanation for the hair growth promotion effect exerted by AAE. PUFA has been reported to reduce UV-induced epidermal thickening [39] and effectively stimulates the duplication of Dermal Papilla cells [29].

**Figure 1:**
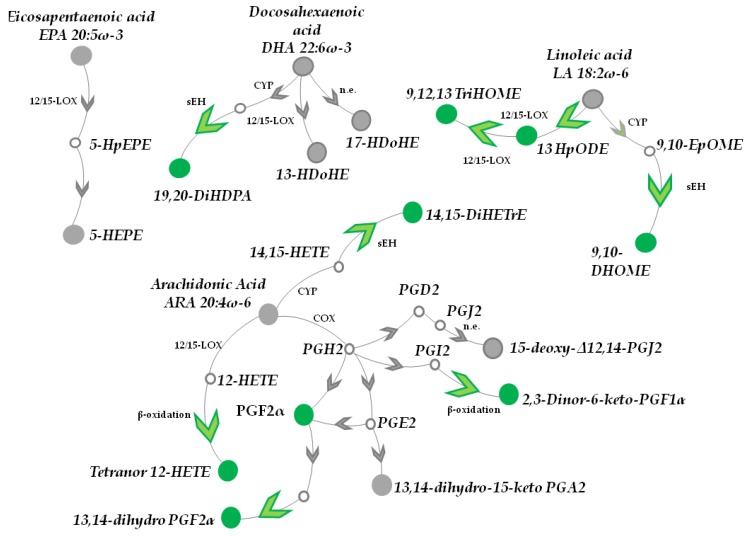
Topical treatment with AAE alters PUFAs metabolome in murine HFs. Schematic cartoon depicting some of the metabolic reactions positively affected by AAE in murine HFs. Green arrowheads indicate reactions stimulated by AAE. Green dots indicate metabolites, whose intracellular concentration resulted increased by AAE. (COX cyclooxygenase; LOX, lipoxygenase; CYP, cytochrome P450; sEH, soluble epoxide hydroxylase; n.e. non enzymatic. The abbreviations of PUFA metabolites as well as their full IUPAC name are shown in Table S1).

C57BL/6 mice were treated topically with a cosmetic foam supplemented either with AAE or with a placebo. Foams were applied on the dorsal skin of 7 weeks old mice (HFs in telogen [40]). We have already shown that the cosmetic foam is able to accelerate telogen to anagen transition in murine HFs and to alter the metabolism of HFs [21]. After 4 weeks of treatment, mice (11 week old) were sacrificed and their dorsal skin excised. HFs were plucked out and their intracellular metabolites extracted. Metabolites of HF cells were identified and measured by DI-FT-ICR mass spectrometry and the levels of each metabolite in AAE and placebo treated HFs were compared. The high mass accuracy (average: 0.087 ppm, Figure 1, Table 1 and Table S1), isotopic distribution and comparison with available standards, ensured confident identification of the discussed metabolites. 

#### 3.1.1. DHA, EPA, LA and α-LA Metabolites

We started looking at metabolites of Docosahexaenoic acid (DHA 22:6ω-3), an essential and very abundant omega-3 fatty acid (Figure 1, Table 1 and Table S1). DHA intracellular levels were not statistically altered by AAE (0.9 ± 0.2 fold, mean ± s.e.m., *p* value > 0.05).

The same was measured for 17-HDoHE (0.95 ± 0.1 fold, *p* value > 0.05) and 13-HDoHE (1.1 ± 0.1 fold, *p* value > 0.05) two products of DHA non-enzymatic oxidation. On the contrary, we measured a statistically significant elevation in 19,20-DiHDPA (1.3 ± 0.1 fold, *p* value < 0.01), a DHA catabolite produced via: (i) CYP-catalysed epoxidation at the DHA omega-3 double bond, followed by (ii) conversion to the vicinal diol by Epoxide Hydrolases (sEH). 

We could as well identify in our metabolic analysis Eicosopentaneoic Acid (EPA 20:5ω-3) and the product of its non-enzymatic oxidation, 5-HEPE (Figure 1, Table 1 and Table S1). However, their intracellular levels (0.9 ± 0.1 fold, *p* value > 0.05 and 1.0 ± 0.1 fold, *p* value > 0.05) were unaltered by the treatment with AAE. Similarly, the intracellular level of the PUFAs Linoleic acid (LA 18:2ω-6) and α Linoleic acid (α LA 18:3ω-3) did not show to be altered by AAE (0.9 ± 0.1 fold, *p* value > 0.05; 1.1 ± 0.1 fold, *p* value > 0.05). 

On the contrary, treatment with AAE increased in vivo the LA catabolites 9,10-DHOME (specific for AAE and absent in HFs treated with placebo) and 9,12,13 TriHOME (1.3 ± 0.1 fold, *p* value < 0.05). 9,10-DHOME originates from 9,10-EpOME, a leukotoxin obtained from LA by the sequential reaction of CYP and sEH. 13-HpODE, another catabolite of LA produced by LOX12/15., resulted increased by AAE treatment (2.3 ± 0.1 fold, *p* value < 0.001) 

#### 3.1.2. ARA Catabolites (CYP and LOX Metabolites)

We then moved to analyse ARA 20:4ω-6 metabolism. Our metabolic analysis could identify ARA (1.0 ± 0.1 fold, *p* value > 0.05) as well as its catabolites Tetranor (12-HETE) (3.4 ± 0.2 fold, *p* value < 0.001) and 14,15 DiHeTre (3.5 ± 0.1 fold, *p* value < 0.001). Only the intracellular levels of the ARA catabolites were increased upon treatment with AAE. In details, Tetranor (12-HETE) is the product of β-oxidation of 12-HETE (the result of ARA oxidation by 12/15-LOX), while 14,15-DiHeTre is the product of the hydrolysis catalysed by sEH of 14,15-HETE an epoxy-eicosatrienoic acid produced by CYP from ARA.

#### 3.1.3. AAE Accelerates Epoxides Conversion into Inactive Diols and β-Oxidation of PUFA Metabolites

The intracellular levels of the most abundant unprocessed PUFAs, ARA, EPA, LA, αLA and DHA were thus not altered by AAE. Since PUFA release from membrane lipids is initiated when a specific stimuli activate Phospholipase A2, our results point toward AAE not affecting Phospholipase A2 activity at least in murine HFs.

On the contrary AAE increased in vivo the intracellular levels of 19,20-DiHDPA, 9,10-DHOME, 14,15-DiHETrE. These PUFA catabolites all share a related origin. They are all diols deriving from the corresponding PUFA epoxides, 19,20-HDPA (Epoxide of DPA), 9,10-epHOME (Epoxide of LA), 14,15-HETE (Epoxide of LA), respectively. In vivo, these epoxides are all generated by CYP epoxidase and are endowed with anti-inflammatory properties [41]. PUFA epoxides are short living and are converted into their inactive diols (among which 19,20-DiHDPA, 9,10-DHOME, 14,15-DiHETrE) by the family of enzyme of soluble epoxidase hydrolases (sEH) [42]. sEH blocks the anti-inflammatory activities of PUFA epoxides promoting their degradation via mitochondrial or peroxisomal β-oxidation.

β-oxidation of PUFA derived metabolites could also be the meaning behind the increase of Tetranor 12-HETE. This metabolite is the major β-oxidation product of 12(S)-HETE a product of ARA metabolism. All together our results seem to depict a scenario where ignition of mitochondrial activity induced by AAE in HFs [21] hijacks many PUFA metabolites toward β-oxidation. PUFA epoxides are thus converted into inactive diols, ultimately altering the overall balance of biologically active lipids and signalling molecules in HFs. 

#### 3.1.4. Prostaglandins

Finally we measure the intracellular levels of prostaglandins. PGD2 and PGE2 have been shown to inhibit and stimulate hair growth, respectively [25,43,44,45] and PGF2 α has recently attracted the interest of dermatologists in virtue of its stimulatory effect on hair growth. Patients affected by glaucoma and receiving PGF2α analogues, claimed side effects such as cutaneous hypertrichosis and hyperpigmentation of eyelashes [32]. PGF2α analogues latanoprost and bimatoprost are being tested in clinical trials for Patterned Hair Loss and Androgenic Alopecia and are showing promising results [4,31].

Analysis of prostaglandins was complicated by their isobaric molecular weights. Notwithstanding, we could measure an increase of the intracellular levels of 2,3-Dinor-6-keto-PGF1α (1.6 ± 0.2 fold, *p* value < 0.05), a major β-oxidation product of the prostacyclin PGI2. On the contrary AAE did not increase the intracellular levels of 15-deoxy-Δ12,14-prostaglandin J2 (1.1 ± 0.1 fold, *p* value > 0.05), nor those of 13,14-dihydro-15-keto PGA2 (1.0 ± 0.1 fold, *p* value > 0.05), both non-enzymatic oxidation products of PGD2 and PGE2, respectively, excluding their involvement in AAE mechanism.

Differently, AAE increased the intracellular level of PGF2alpha (1.6 ± 0.1 fold, *p* value < 0.05) and 13,14-dihydro PGF2α (2.3 ± 0.2 fold, *p* value < 0.001) a metabolite resulting from 13,14-dihydro-15 keto PGF2α.

#### 3.1.5. AAE Selectively Activates Prostanoid Metabolism in HF Cells

The increase in PGF2α could be explained with a stimulatory activity exerted by AAE on COXs. However, in our opinion this seems not be the case considering that PGD2, PGE2, PGI2 and PGF2α are all produced starting from Prostaglandin H2 (PGH2), the main product of COXs activity on ARA. In virtue of this, AAE should have up-regulated all the prostaglandins. We believe that the selective increase in PGF2α production might reflect the selectivity of AAE for hair bulge cells of the HFs, we already documented [21]. Enzymes producing PGF2α or converting PGE2 into PGF2α are preferentially expressed in bulge cells and in ORS originating from them and only weakly in HM and dermal papilla [23]. We have already shown that AAE seems to activate mitochondrial activity specifically in hair bulge and ORS keratinocytes [21] sparing HM cells of the dermal papilla. It is probably this selectivity of AAE toward specific HFs cells, to allow the increase of PGF2α intracellular levels, leaving unaltered those of the other prostanoids. Despite this is a likely hypothesis, further experiments will be necessary to confirm it.

### 3.2. AAE Protects Murine HFs from Taxane Induced Follicular Dystrophy

When cultivated in appropriate growth conditions, excised HFs may sustain in vitro hair shaft extension. The resulting ex vivo culture preserves several biological properties of the HFs and respond to chemotherapy agents showing some of the key features of CIA. Here we used skin biopsies of 12 week old C57BL/6 mice (anagen phase, [40]) to perform an ex-vivo incubation of HFs in the presence of paclitaxel (700 nM) or docetaxel (700 nM) for 7 days. We further tested if 400 mg/L of AAE (AAE active dose for hair promoting effect [20]) or the corresponding amount of vehicle (DMSO), was able to reduce the HF dystrophy induced by the two taxanes. After 7 days of incubation, skin biopsies were processed for histology and classified following morphological criteria [12,40,46].

**Figure 2:**
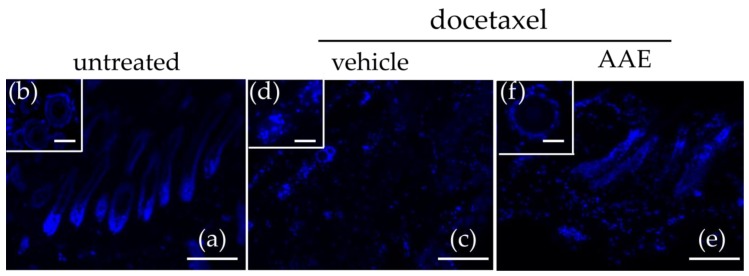
AAE protects murine HFs from taxanes induced dystrophy. 12 week old C57BL/6 mice were sacrificed and their skin biopsies incubated in vitro in the absence or in the presence of Docetaxel (700 nM), AAE (400 mg/L) or vehicle. Upon 7 days of ex-vivo culturing, biopsies were fixed and processed for histology. Nuclei were stained with DAPI and HFs classified following morphological criteria. (**a**,**b**) Untreated HFs in late Anagen phase; (**c**,**d**) HFs treated with Docetaxel showing severe signs of follicular dystrophy (**e**,**f**) HFs of mice treated with docetaxel in the presence of AAE appearing less damaged by the treatment with the taxane. Scale bars correspond to 50 μm.

In the absence of any treatment, ex-vivo cultures of HFs were mostly in Anagen (Figure 2a). This was expected, since the hair cycle of C57BL/6 mice is fully synchronized and at week 12, all the dorsal skins of mice belonging to this strain present HFs in anagen, a phase documented to last until week 15 [40]. In sagittal sections, untreated HFs appear as ‘well developed’ terminal follicles, with HBs almost totally located in the middle of subcutis, close to the *panniculus carnosus* (the subcutaneous muscle layer) (Figure 2a). Moreover, transversal section clearly show fully developed ORS and IRS, with no signs of apoptosis or fibrous tracts (Figure 2b).

Treatment with taxanes (both Docetaxel (Figure 2c,d) and Paclitaxel (data not shown)) massively affected HFs, that showed clear signs of chemotherapy induced damage. In sagittal skin sections, we did not detect terminal healthy follicles but, on the contrary, mostly degenerated follicular units and HFs remnants (Figure 2c). Transversal sections showed dispersed fibrous tracts and the few follicles visible presented loss of ORS layer and severe IRS wrinkling (Figure 2d).

When HFs were treated with taxanes in the presence of AAE, HFs appeared much less damaged by the treatment (Figure 2f). Their morphology and average length were preserved as well as their location close to the subcutis. Moreover, in transversal sections no evident signs of ORS and IRS damage was visible (Figure 2f).

### 3.3. AAE Preserves Keratin Production in Murine HFs Treated with Taxanes

**Figure 3:**
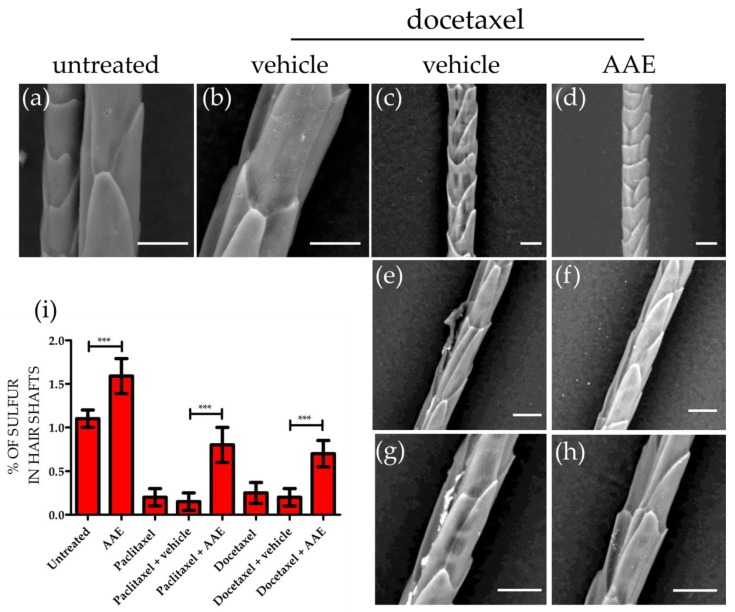
AAE preserves keratin content in murine HFs treated with taxanes. Hair shafts plucked out from mice biopsies treated as in Figure 2 were analysed by SEM-EDX. Morphology of hair shafts extracted from untreated skin biopsies (**a**) or treated with docetaxel (700 nM; **b**,**c**,**e**,**g**) and showing signs of hair shaft damage. Morphology of hair shafts extracted from HFs treated with Docetaxel in the presence of AAE (**d**,**f**,**h**) showing signs of hair shaft protection exerted by AAE (Scale bars correspond to 10 μm). (**i**) SEM-EDX quantitative analysis indicates a decrease in Sulphur content in hairs shafts extracted from HFs treated with taxanes. In the presence of AAE the Sulphur content of hair shafts is partially preserved (mean ± s.e.m.; n = 8, *** *p* < 0.001).

To confirm the protective effect exerted by AAE against taxane induced dystrophy, hair shaft microstructure and keratin content of HFs were evaluated by SEM-EDX [36]. To analyse the hair shaft extension occurred only during the period of the in vitro incubation, we evaluated only hair shafts section located in the HFs or closest to them. Differently from untreated HFs (Figure 3a), hair shafts of HFs treated with Docetaxel (Figure 3b,c,e,g) or Paclitaxel (data not shown) appeared frequently damaged and when analysed by EDX presented a drastic decrease in Sulphur content (Figure 2i). Since Cystine, Methionine, Cysteine and Cysteic acid are abundant amino acids of hair keratins, the percentage of Sulphur in the hair shaft can be considered a measure of keratine amount. We have already shown [21] that AAE is able to increase Sulphur content in hair keratins. Here, when the incubation with taxanes was performed in the presence of AAE (400 mg/L), hair shaft showed microstructure and Sulphur content similar to that of untreated HFs (Figure 2d,f,h,i).

Our morphological data and SEM-EDX analysis thus suggest that 400 mg/L AAE were able to protect, at least in vitro, murine HFs from taxane induced dystrophy and to preserve in vitro keratin production.

## 4. Discussion

Annurca Apples have been showing their potential as nutraceuticals in many human conditions and pathologies. The plethora of different biological contexts in which AAE is active must be attributed to the hundreds of different metabolites it contains [47]. The resulting molecular synergism allows AAE to act as antioxidant, as modulator of lipid and cholesterol anabolism as well as against stress and aging [19,48,49,50].

In our recently published clinical trial [20], we have proved that the consumption of AAE is able to promote hair growth in healthy subjects and to increase hair number, hair weight and keratin content in humans. Using a murine model and high resolution mass spectrometry we have as well disclosed some of the molecular details behind AAE hair promoting effect [21]. AAE inhibits glutaminolysis, PPP as well as glutathione, citrulline and nucleotide synthesis switching intracellular HFs metabolism towards mitochondrial respiration and β-oxidation. As result of this drastic metabolic re-programming, amino acids are spared from being oxidized and are ultimately kept available for keratin biosynthesis [21].

Herein, our analysis is further extended to show the effect exerted by AAE on PUFAs, a class of lipids involved in HF signalling and homeostasis [23,24]. By monitoring the change in the intracellular levels of PUFA and of their metabolites (Figure 1, Table 1 and Table S1) we could show that AAE hijacks most of them toward β-oxidation. AAE induces PUFA epoxides conversion into inactive diols as well as prostanoids conversion into inactive catabolites. As result, AAE promotes their usage as β-oxidation substrates, altering the overall balance of these biologically active lipids in HFs. Interestingly, the prostanoid PGF2α a potent hair growth promoter is one of the PUFA metabolite that is selectively increased by AAE in HFs and likely one of the molecule contributing to the hair promoting effect of AAE.

The metabolic switch induced by AAE in HFs has interesting consequence in terms of compatibility of AAE with chemotherapy regimens. By blocking glutaminolysis, AAE impairs the synthesis of nitrogen containing bases and of deoxy-nucleotides. The absence of metabolites necessary for DNA replication and RNA production explains why AAE does not promote cancer cell growth and mitosis [12]. Moreover, by reducing nucleotide synthesis, AAE makes HFs resistant to chemotherapy agents like taxanes, that selectively inhibit mitotic and highly proliferating cancer cells. This peculiar mechanism, together with the anticancer property we have described for AAE in colon rectal cancer cells [12], suggests that AAE could represent a safe nutraceutical option for hair growth and an interesting alternative to synthetic drugs for treating CIA.

Indeed, by using ex-vivo cultures of HFs, we prove that 400 mg/L AAE (an amount corresponding to the daily dosage recommended for consumption in humans) reduced hair bulb dystrophy and efficiently preserved keratin production in murine HF treated with docetaxel and paclitaxel, two taxanes commonly causing irreversible alopecia in breast cancer patients. Ex-vivo cultures of human HFs as well as animal models of CIA, will help in the future to finally clarify if AAE can be elected as candidate nutraceutical against CIA.

## Figures and Tables

**Table 1:** Fold induction for the indicated metabolites measured upon in vivo treatment with AAE.

PUFA	Metabolite	Fold Change ^1^	PUFA	Metabolite	Fold Change ^1^
ARA			DHA		
	ARA 20:4ω-6 *	1.0 ± 0.1		DHA 22:6ω-3 *	0.9 ± 0.2
	Tetranor 12-HETE	3.4 ± 0.2		17-HDoHE	1.0 ± 0.1
	14,15-DiHETrE	3.5 ± 0.1		13-HDoHE	1.1 ± 0.1
	2,3-Dinor-6-keto-PGF1α	1.6 ± 0.2		19,20-DiHDPA	1.3 ± 0.1
	15-Keto-13,14-dihydroPGA2	1.0 ± 0.1			
	PGF2α	1.6 ± 0.1	α-LA		
	13,14-dihydro-PGF2α	2.3 ± 0.2		α-LA 18:3ω-3 *	1.1 ± 0.1
	15-deoxy-Δ12,14-PGJ2	1.1 ± 0.1	LA		
				LA 18:2ω-6 *	0.9 ± 0.1
EPA				9,10-DHOME	specific for AAE
	EPA 20:5ω-3 *	0.9 ± 0.1		13-HpODE	2.3 ± 0.1
	5-HEPE	1.0 ± 0.1		9,12,13-TriHOME	1.3 ± 0.1

^1^ (n = 15. Shown is mean ± s.e.m.); * indicates unprocessed PUFA. The abbreviations of PUFA metabolites as well as their full IUPAC names are shown in Table S1.

## Supplementary Materials

The following are available online at http://www.mdpi.com/2072-6643/10/11/1808/s1, Figure S1: Partial least squares-discriminant analysis (PLS-DA) of HFs metabolites determined by FT-ICR–MS, Table S1: Identification of metabolites in HFs determined by DI-FT-ICR-MS.
