# Supplementary material for: Annurca Apple Polyphenols Protect Murine Hair Follicles from Taxane Induced Dystrophy and Hijacks Polyunsaturated Fatty Acid Metabolism toward β-Oxidation
Source: Nutrients. 2018 Nov 20;10(11):1808. doi: 10.3390/nu10111808 (PMC6267362; doi:10.3390/nu10111808)
Supplement: Supplementary file 1 [file nutrients-10-01808-s001.pdf]

Supplementary material for the manuscript

Annurca Apple Polyphenols protect murine Hair Follicles from Taxane Induced Dystrophy and hijacks PUFA metabolism toward β-oxidation.

Gennaro Riccio [1†], Eduardo Sommella [2†], Nadia Badolati [1], Emanuela Salviati [2,3] , Sara Bottone [1] , Pietro Campiglia [2], Monica Dentice [4], Giancarlo Tenore [1], Mariano Stornaiuolo [1,*] and Ettore Novellino [1,*]

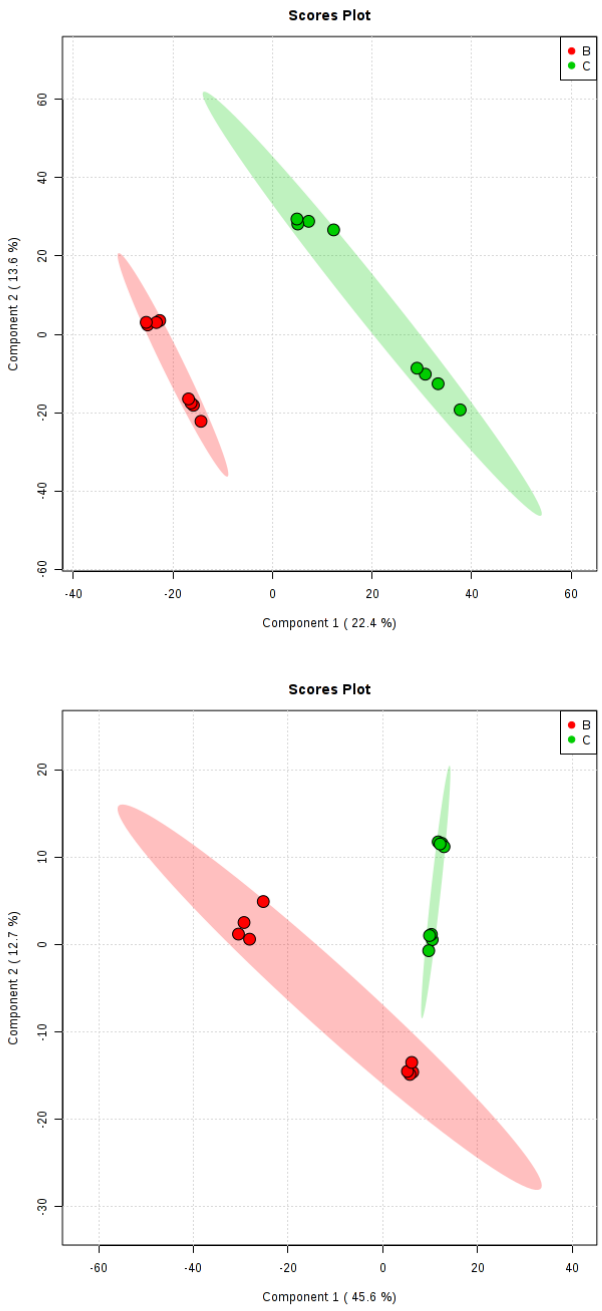

Figure S1 a (top), b (bottom): Partial least squares-discriminant analysis (PLS-DA) of HFs metabolites determined by FT-ICR–MS, the two dimensional score plots show clustering and separation between AAE treated mice (green symbols) and Placebo (red symbols). Ellipses represent 95% confidence intervals.

IUPAC NAME	Metabolite	m/z	Δm/z [ppm]	Ions
**(5Z,8Z,11Z,14Z)-icosa-5,8,11,14-tetraenoic acid**	ARA 20:4ω-6	327.2295	0.236	[M+Na]+
**(4Z,6E,8S,10Z)-8-hydroxyhexadeca-4,6,10-trienoic acid**	Tetranor 12-HETE	289.1774	0.023	[M+Na]+
**(5Z,8Z,11Z)-14,15-dihydroxyicosa-5,8,11-trienoic acid**	14,15-DiHETrE	361.235	0.018	[M+Na]+
**5-[(1R,2R,3R,5S)-3,5-dihydroxy-2-[(1E,3S)-3-hydroxyoct-1-en-1-yl]cyclopentyl]-4-oxopentanoic acid**	2,3-Dinor-6-keto-PGF1 α	365.1936	0.323	[M+Na]+
**(5Z)-7-[(1R,5S)-2-oxo-5-(3-oxooctyl)cyclopent-3-en-1-yl]hept-5-enoic acid**	15-Keto-13,14-dihydroPGA2	357.2037	-0.124	[M+Na]+
**(5E)-7-[(1R,2R,3R,5S)-3,5-dihydroxy-2-[(1E,3S)-3-hydroxyoct-1-en-1-yl]cyclopentyl]hept-5-enoic acid**	Prostaglandin F2 α	377.2300	0.171	[M+Na]+
**(5E)-7-[3,5-dihydroxy-2-(3-hydroxyoctyl)cyclopentyl]hept-5-enoic acid**	13,14-dihydro-PGF2α	357.2612	0.038	[M+Na]+
**(Z)-7-[(1S,5E)-5-[(E)-oct-2-enylidene]-4-oxocyclopent-2-en-1-yl]hept-5-enoic acid**	15-deoxy-Δ12,14-PGJ2	339.1932	0.022	[M+Na]+
**(5Z,8Z,11Z,14Z,17Z)-icosa-5,8,11,14,17-pentaenoic acid**	EPA 20:5ω-3	325.2050	0	[M+Na]+
**(6E,8Z,11Z,14Z,17Z)-5-hydroxyicosa-6,8,11,14,17-pentaenoic acid**	5-HEPE	341.2088	0.289	[M+Na]+
**(4Z,7Z,10Z,13Z,16Z,19Z)-docosa-4,7,10,13,16,19-hexaenoic acid**	DHA 22:6ω-3	351.2295	0.113	[M+Na]+
**(4Z,7Z,10Z,13Z,15E,19Z)-17-hydroxydocosa-4,7,10,13,15,19-hexaenoic acid**	17-HDoHE	367.2245	0.416	[M+Na]+
**(3Z,6E,8E,12Z,15Z,18Z)-docosa-3,6,8,12,15,18-hexaen-10-ol**	13-HDoHE	337.2503	-0.099	[M+Na]+
**(4Z,7Z,10Z,13Z,16Z)-19,20-dihydroxydocosa-4,7,10,13,16-pentaenoic acid**	19,20-DiHDPA	385.235	0.393	[M+Na]+
**(9Z,12Z,15Z)-octadeca-9,12,15-trienoic acid**	α-LA 18:3ω-3	301.2138	0.023	[M+Na]+
**(9Z,12Z)-octadeca-9,12-dienoic acid**	LA 18:2ω-6	303.2295	-0.017	[M+Na]+
**(12Z)-9,10-dihydroxyoctadec-12-enoic acid**	9,10-DHOME	313.2384	-0.098	[M-H]-
**(9Z,11E,13S)-13-hydroperoxyoctadeca-9,11-dienoic acid**	13-HpODE	335.2193	-0.017	[M+Na]+
**(9S,10E,12S,13S)-9,12,13-trihydroxyoctadec-10-enoic acid**	9,12,13-TriHOME	353.2299	-0.094	[M+Na]+

Table S1: Identification of metabolites in HFs determined by DI- FT-ICR-MS
